# Patterns of Distribution and Spatial Indicators of Ecosystem Change Based on Key Species in the Southern Benguela

**DOI:** 10.1371/journal.pone.0158734

**Published:** 2016-07-21

**Authors:** Katherine E. Watermeyer, Laurence Hutchings, Astrid Jarre, Lynne J. Shannon

**Affiliations:** Marine Research Institute (Ma-Re) & Department of Biological Sciences, University of Cape Town, Cape Town, South Africa; Department of Agriculture and Water Resources, AUSTRALIA

## Abstract

Several commercially and ecologically important species in the southern Benguela have undergone southward and eastward shifts in their distributions over previous decades, most notably the small pelagic fish sardine *Sardinops sagax* and anchovy *Engraulis encrasicolus*. Understanding these changes and their implications is essential in implementing an ecosystem approach to fisheries in the southern Benguela and attempting to appreciate the potential impacts of future environmental change. To investigate possible impacts of these shifts at an ecosystem level, distribution maps for before (1985–1991), during (1997–2000) and after (2003–2008) the shift in small pelagic fish were constructed for 14 key species from catch and survey data, and used to calculate spatial indicators including proportion east and west of Cape Agulhas, relative overlap in biomass and area, index of diversity, connectivity. Potential interactions on the south and west coasts were also compared. For several species (redeye; chub mackerel; kingklip; chokka squid; yellowtail), previously unidentified increases in the proportion of biomass east of Cape Agulhas were shown to have occurred over the same period as that of small pelagic fish, although none to the same degree. On average, overlap with small pelagic fish increased over time and overall system connectivity was lowest in the intermediate period, possibly indicating a system under transition. Connectivity declined over time on the west coast while increasing on the east coast. Distributions of other species have changed over time, with the region east of Cape Agulhas becoming increasingly important in terms of potential trophic interaction. Variations in distribution of biomass and structural complexity affect the trophic structure and hence functioning of the system, and implications should be considered when attempting to identify the possible ecosystem impacts of current and future system-level change.

## Introduction

An understanding of the functioning of an ecosystem is not achievable without knowledge of the interactions involved. One way in which this knowledge can be expanded is by observing any changes in the relative distributions of trophically linked species over time. Monitoring of past and current patterns becomes of even greater importance as climate change impacts become more apparent. Changes in migration and distribution ranges, as well as density distribution, are all known to be strongly influenced by environmental fluctuations [1] and have already been shown in multiple regions and species globally [2–6].

**Fig 1:**
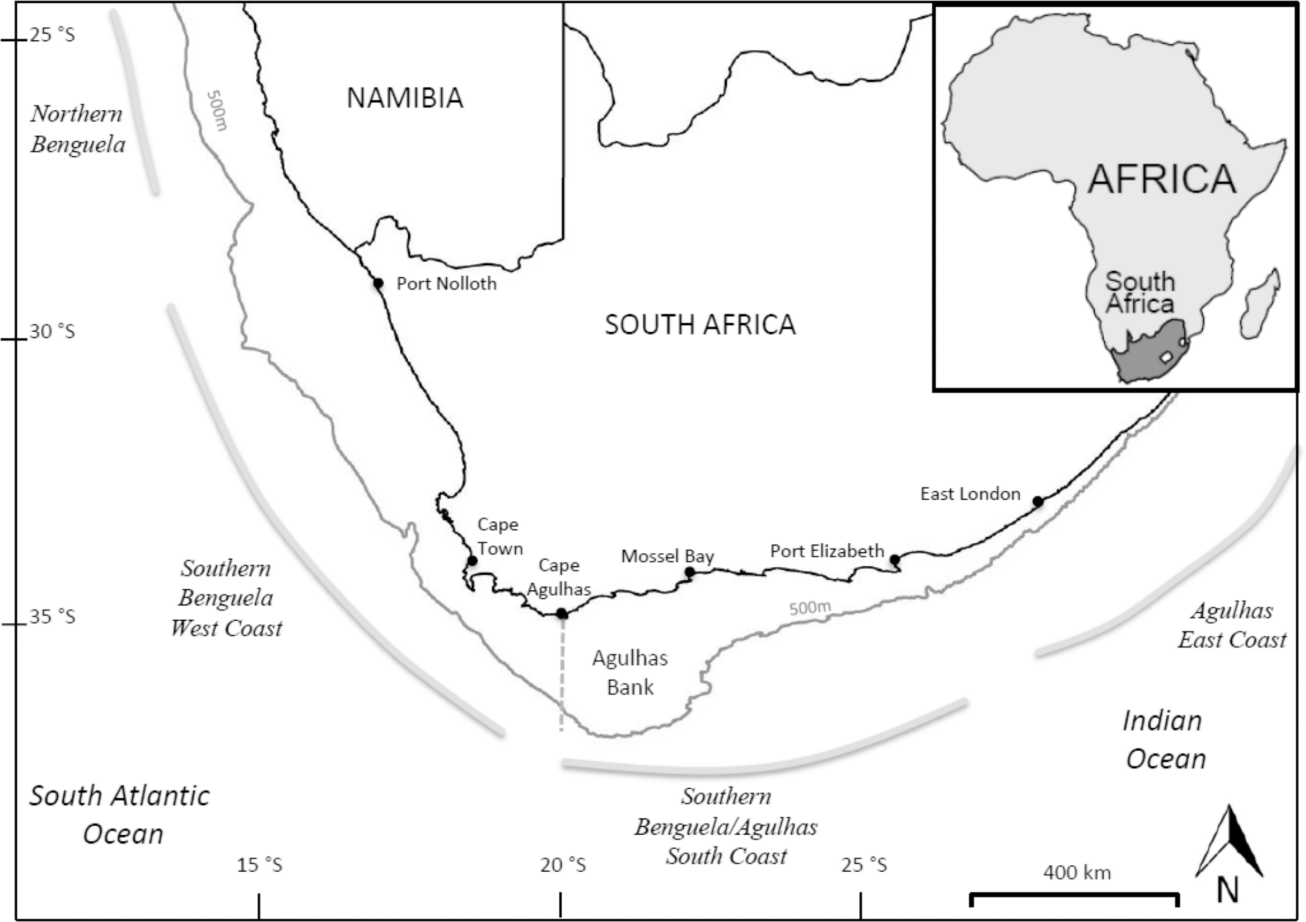
The southern Benguela with west and south coasts divided at Cape Agulhas. The shelf-edge is indicated by the 500m isobath [9].

The southern Benguela is an eastern boundary current system extending from the Lüderitz upwelling cell at 26°S in the north around the coast of South Africa to East London at 28°E (Fig 1). The system is comprised of two physically and functionally different regions (i) the west coast, a classic wind-driven upwelling system, and (ii) the south coast which includes the Agulhas Bank and as a result, has characteristics of both upwelling and shelf systems [7,8]. Many species, particularly the small pelagic fish sardine *(Sardinops sagax)* and anchovy *(Engraulis encrasicolus)* have a migratory life history: adults spawn on the Agulhas bank and eggs and larvae are transported to the more productive west coast where recruitment and feeding takes place. Spawners then return to the south coast to complete the cycle. The system supports a relatively high fish biomass, which in turn supports a number of commercially important fisheries, the two largest in terms of catch and in economic value being the demersal trawl fishery, targeting deep and shallow water hake (*Merluccius paradoxus* and *M*. *capensis*), and the small pelagic purse-seine fishery.

The concept of regime shifts, or a sudden and widespread change from one stable system state to another, has become more widely used in marine science as longer datasets become available for analysis [10,11]. When a number of physical and biological timeseries from the southern Benguela were analysed using methods to detect regime shifts, many displayed shifts between the mid-1990s and the early 2000s. Based on these findings, an ecosystem-level regime shift is thought to have occurred in the southern Benguela in the early 2000s [12–14].

One of the most apparent changes is in the distribution of small pelagic fish (hereafter referring to sardine and anchovy unless otherwise specified), whose relative abundance declined on the west coast and increased on the south coast (east of Cape Agulhas, see Fig 1) in the late 1990s [15,16]. Small pelagic fish are trophically very important within the system and can exert both bottom-up and top-down effects on other species in what is known as wasp-waisted trophic control [17,18]. It therefore seems likely that this eastward increase in biomass, particularly when combined with the shifts in other variables that have since been identified, would have caused regional changes in the spatial interactions with trophically linked species and thus influenced the trophic functioning of the system. Observed trends in seabird abundance, distribution and breeding success since the mid-1990s have already been linked to the concurrent changes in small pelagic fish distribution [19]. Substantial declines in African penguins and gannet populations over the early 2000s, for example, have been related to the availability of small pelagics as prey, as has the increased abundance in Cape cormorants and swift terns on the south coast [19–22].

Without knowledge of the interactions within a system, an effective application of an ecosystem approach to fisheries (EAF) is likely unattainable. Observing patterns in the relative distributions of trophically linked species, and monitoring change over time, can be a useful means of understanding system structure and any change it may undergo [2,4,23,24]. It can also allow spatial ecosystem indicators to be calculated. Indicators are an important component of applying an EAF and allow a large amount of data to be distilled into more easily communicable values, useful for monitoring temporal and spatial change, as well as evaluating management objectives [25–28].

Distribution maps for key species in the southern Benguela were previously constructed for the 1980s and 1990s [29]. These maps aggregated over that entire period (1980s and 1990s) were then used to calculate a measure of spatial interaction between species and indicators of ecosystem state [24,30]. However, because ecosystem-level changes, including substantial distributional changes in key forage fish species (sardine and anchovy), have been identified as occurring over the late 1990s to early 2000s, previous findings are unlikely to be representative of the system beyond the mid-1990s.

Distribution maps were plotted for key species in the southern Benguela and compared over relevant time periods selected based on previously identified shifts, with a focus on changes in small pelagic fish distribution. Any changes in the distribution of other species or in spatial indicators over time can be used to identify possible changes in the level of interspecific interaction and increase our understanding of the changes at an ecosystem level in the context of future effects of climate change.

## Methods

### Time periods

The average spatial distribution during different periods was used as a means of examining change over time, based on the concept of regime shifts. System level shifts have been previously identified across biological and physical elements of the southern Benguela, the first in the early to mid-1990s and the second in the early 2000s [12,31,32], coinciding with a peak in small pelagic fish biomass. Based on these shifts, three time periods were chosen as representing different ecosystem states to be compared. Period 1 was chosen as 1985–1991, representing the system before the increased abundance and eastward shift in sardine and anchovy; Period 2 as 1997–2000, an intermediate/transition phase; and Period 3, during which both sardine and anchovy were predominantly found east of Cape Agulhas, as 2003–2008. To create a clear snapshot of the specified periods and the ecosystem states they represent, the years between these selected periods (1992–1996 and 2001–2002) were omitted.

### Distributions

Data from either scientific surveys or commercial catch records were used to plot distribution maps. When distributions have been plotted previously [29], all available data sources were combined into a single distribution map. This requires a number of assumptions to be made regarding the ability of various data sources to represent a particular species’ distribution and the possible biases involved. For example, both commercial catch data and survey data are by definition more representative of the species targeted by that fishery or for which the survey is designed, and are likely to underrepresent other species. To combine the two data sources one would either need to assume equally accurate representation of the species in question by both, or decide on a weighting to the importance or representativeness of each. The aim in the current study was to detect change over time rather than to produce the most accurate distribution map possible. Therefore, in this context, rather than combine all information, a single data source was selected for each species as the most representative and best suited to reflecting temporal change for that species (see details below). Resultant distributions, while not assumed to be complete, are representative and appropriate given the nature of the study.

A total of 14 key species were examined: anchovy; sardine; round herring *(Etrumeus whiteheadi)*; Cape hake *(Merluccius capensis)* and *(M*. *paradoxus);* horse mackerel *(Trachurus trachurus capensis)*; chub mackerel *(Scomber japonicus)*; kingklip *(Genypterus capensis);* chokka squid *(Loligo vulgaris reynaudi);* snoek *(Thyrsites atun);* silver kob *(Argyrosomus inodorus);* yellowfin tuna *(Thunnus albacares);* yellowtail *(Seriola lalandi)* and geelbek *(Atractoscion aequidens)*. To choose the data source most representative of each, plots for each species for Period 3 (2003–2008) were constructed from all available data sources. Based on these maps and discussions with relevant experts for each species (scientists at the relevant South African academic and government institutions), a single data source was selected as most representative and most likely to illustrate change over time for each species (Table 1). To allow comparison between data sources that have different spatial scales, all data were joined to a 10’x10’ reference grid. All distributions were plotted using ArcGIS v 10.1.

**Table 1:** Data sources and units used to plot distributions for each species.

Species	Data source	Units
**Sardine** *(Sardinops sagax)*	Pelagic surveys^*^	g/m^2^
**Anchovy** *(Engraulis encrasicolus)*	Pelagic surveys	g/m^2^
**Round herring** *(Etrumeus whiteheadi)*	Pelagic surveys	g/m^2^
**Shallow-water hake** *(M*. *capensis)*	Demersal surveys**	kg/hr
**Deep-water hake** *(M*. *paradoxus)*	Demersal surveys	kg/hr
**Horse mackerel** *(Trachurus trachurus capensis)*	Demersal surveys	kg/hr
**Chub mackerel** *(Scomber japonicas)*	Demersal surveys	kg/hr
**Kingklip** *(Genypterus capensis)*	Demersal commercial	kg/hr
**Chokka** *(Loligo vulgaris reynaudi)*	Demersal commercial	kg/hr
**Snoek** *(Thyrsites atun)*	Demersal commercial	kg/hr
**Yellowfin tuna** *(Thunnus albacares)*	Longline	kg/1000 hooks
**Silver kob** *(Argyrosomus inodorus)*	Line fishery	kg/day
**Yellowtail *(****Seriola lalandi)*	Line fishery	kg/day
**Geelbek *(****Atractoscion aequidens)*	Line fishery	kg/day

### Data sources and plotting

#### Survey data

Annual pelagic hydro-acoustic surveys of principal small pelagic fish species (anchovy, sardine and round herring), comprised of a summer (November) spawner biomass and a winter (May) recruitment survey, have produced reliable data for the southern Benguela since 1984 [33]. Survey design and methods have been thoroughly described in [33] and [34]. Although initially designed to monitor anchovy, the abundance and biomass estimates from these surveys are now an essential input into the management process and serve as a basis for total allowable catch (TAC) recommendations for both sardine and anchovy fisheries [33,35]. The area covered by the pelagic surveys initially extended from either the Orange River (May survey) or Hondeklip Bay (November survey) on the west coast to Port Alfred on the south coast (Fig 1), but in recent years has extended further east. The time series has been revised to take into account changes in survey equipment and an increased understanding of possible sources of error in earlier estimates [35,36]. Pelagic survey data from both the May and November annual surveys combined were assumed as the most representative of the distribution of the small pelagic sardine, anchovy and round herring and were used to construct maps and in analysis for these species.

Positions of May and November pelagic survey intervals and their corresponding densities of sardine, anchovy and round herring, including zero values, were plotted and joined to the 10’x10’ reference grid. Average annual density per grid cell was calculated for each period. Although May and November surveys estimate recruit numbers and spawner biomass respectively, and thus reflect different stages of the life cycle and the associated differing distributions, the data from both surveys were combined. Because the aim of this investigation was to increase understanding of ecosystem function and how changes in distribution may have affected it, the point of interest lies in the availability of food to predators. Therefore both recruits and adult fish were considered. Data from both surveys were combined assuming an equal weighting. To avoid skewing the results due to the extension of sampling effort along the east coast during the more recent periods, an easterly limit on acceptable data points was set corresponding to the most easterly extent of surveys during Period 1.

Demersal biomass surveys, as described by [37], are also conducted around the coast of South Africa. Surveys are designed and used primarily for the estimation of hake biomass to inform the management of the hake-directed trawl fishery. Cruises have been conducted on the west coast (Orange River to Cape Agulhas) biannually from 1986–1990, after which the winter cruise was discontinued [32]. On the south coast (Cape Agulhas to Port Alfred) surveys were conducted annually in spring from 1986–1990, and subsequently in both spring and autumn [38]. Demersal biomass survey data from all available cruises combined were assumed to be the best descriptor of hake, horse mackerel, and chub mackerel distribution.

Data were excluded where trawl duration was less than 15 minutes or the trawl location north of the Orange River. Survey catches, including zero values, and minutes per trawl were converted to kg/hr. Catches and trawl duration were plotted and joined to the reference grid. The average annual catch in kg/hr per reference grid cell was then calculated for each period.

#### Commercial data

A demersal trawl fishery has been operating off South Africa since the early 1900s [39], initially targeting Agulhas and West Coast sole *(Austroglossus pectoralis)* and *(A*. *microlepis)*, but only targeting hake after the First World War. Catch data are provided in the form of catch weight (kilograms) and duration of trawl per 20’x20’ grid cell. Trawl data were taken as representative of kingklip, chokka squid and snoek distribution. To allow commercial inshore and offshore trawl data already assigned to a 20’x20’ grid to be displayed on the 10’x10’ reference grid, each reference grid cell was assigned one quarter of the catch of the 20’x20’ cell within which it was situated. The average annual catch in kg/hr per reference grid cell was then calculated.

South Africa’s line fishery is one of its oldest, with the boat-based fishery well-established by the mid-1800s. The fishery currently operates around the whole coastline on the continental shelf [40]. Catch and effort data are in the form of catch weight (kg) and catch days reported on a 5x5 minute grid. Line fishery data were used to produce maps for silver kob, yellowtail and geelbek. The 5x5 minute grid was joined to the reference grid and the catch and number of catch days per grid cell summed and used to calculate the average annual catch per fishing day per cell during each period. Data for kob in the linefishery do not distinguish between species, however their distribution is limited to the area between Cape Point and the Kei River mouth (28.37°E) on the south-eastern coast of South Africa (C. Attwood, Marine Research Institute, University of Cape Town). Any records east of this point were excluded from analyses, and all those remaining were assumed to consist of primarily Silver Kob.

An experimental longline fishery for tuna in the southern Benguela began in 1997, and has been operating as a commercial fishery since 2005 [41]. Boats report catch weight (kg) and the number of hooks per set, as well as the start and end positions of the line. Data from this fishery was used as the basis for the yellowfin tuna distribution. Start positions were plotted and the average annual catch in kg/1000 hooks for each reference grid cell during each period calculated.

For all data sources, any data points that fell obviously outside of possible sampling areas (i.e. on land/ far outside of known sampling area) were excluded from analyses. To allow for uncertainty in areas of low density/ catch rate, cells representing the lowest 5% of all distributions in each period were removed, thus the final map used in analysis only represents the core 95% of the distribution.

### Analysis

Distribution maps were used to calculate the following indicators. Indicators i–iv relate to ecosystem state, whereas v is an indicator of pressure.

1. The proportion of biomass found east and west of Cape Agulhas for each species in each period. To test for differences in the observed proportions in each period a beta regression model with a logit link function was fitted to the observed proportions, with period as an explanatory variable. This was done using the betareg package in R [42].
2. The relative overlapping areas and biomass (ROA and ROB) between species
  $${ROA}_{a,j}=\frac{\left(A_{a,j}\cap A_{b,j}\right)}{A_{a,j}}$$
  where a and b are the trophically related species, j is the period and ∩ symbolises the intersect between the two [24,30]. Similarly, the proportion of total biomass overlapping was calculated (ROB). When calculating overlaps between species with distributions based on demersal survey data (hake, horse mackerel and chub mackerel) and small pelagic fish (based on pelagic survey data), only the November spawner biomass pelagic survey data were used for the overlaps due to the timing of the surveys: since 1990, no winter demersal surveys have been conducted. A more accurate overlap could therefore be obtained by excluding the winter pelagic survey from the analysis. For all other species, based on year round commercial data, overlaps were calculated using combined May and November pelagic survey data for small pelagic fish species.
  The averages of ROA and ROB between all species where a trophic relationship (predation or competition) exists (Table 2) were taken as a measure of overall ecosystem connectivity for that period [30]. Although ‘connectivity’ is used in trophic models to refer to the degree of trophic linkage within the system, the term can also be applied to a range of factors relating to states of physical or trophic and literal or potential connectedness, or degree of interaction within systems or between components of those systems, e.g. [43–45]. Here, ‘connectivity’ refers to the average degree of physical overlap between species and thus the potential for interaction [30].
  Relationships were based on those identified by [29], initially derived from trophic relationships in the southern Benguela [46]. Those for geelbek and yellowtail were added based on dietary literature [47–49] and discussion with DAFF scientists. All relationships involving yellowfin tuna were excluded from this calculation, as there are no data for this species during Period 1 (1985–1991). Differences in the degree of overlap over time were again tested using a beta regression model with a logit link function fitted to the observed proportions, with period as an explanatory variable [42].
3. An index of spatial biodiversity (ISB_j_) was calculated for each period (j), based on mapped species only, and excluding yellowfin tuna as no data are available for this species for Period 1. Maps showing the number of species out of the possible 13 found in each cell for each period were also generated. ISB_j_ was calculated as the average proportion of total possible species *S* found in any grid cell during period j according to:
  $${ISB}_{j}=\sum_{g=1}^{n}\left(s_{g,j}/S\right)\times (100/n)$$
  where *n* is the total number of cells with observations and *s* is the number of species in cell *g* [30]. The ISBj indicator has some drawbacks, namely that different patterns can give the same overall average result, and it may not be suited to intersystem comparisons without consideration of the implications of the species included when calculating the index [30]. It does, however, allow for an exploration of possible ecosystem level change over time, and thus is considered suitable for this application (see discussion). Differences in ISB between periods were again tested using a beta regression model with a logit link function fitted to the observed proportions and period as an explanatory variable [42].
4. The proportion of effort east and west of Cape Agulhas was calculated for each data source, in units of survey intervals (pelagic survey), trawl minutes (demersal survey), fishing hours (demersal commercial), hooks set (longline) and catch days (linefishery). These proportions were averaged overall as well as for all commercial data and all survey data, for each period.

**Table 2:** Trophic relationships between species examined, represented qualitatively as either strong (P or C) or moderate (p or c) predation and competition respectively. Weak relationships or occasional predation are not included. Updated from [24].

Prey\ predator	Sd	An	Rd	Mc	Mp	Hm	Cm	Kk	Ck	Sk	Sn	Yf	Yt	Gb
**Sardine (Sd)**	-	C	C	pc	pc	pc	pc		pc	P	Pc		P	P
**Anchovy (An)**	C	-	C	pc	pc	pc	pc		p	P	Pc		P	P
**Round herring (Rd)**	C	C	-	pc	pc	pc	pc		p		Pc			
***M*. *capensis* (Mc)**	c	c	c	P	pc	c	c	Pc	Pc		pc	pc		
***M*. *paradoxus* (Mp)**	c	C	c	pc	P	c	c	Pc	Pc		pc	pc		
**Horse mackerel (Hm)**	c	C	c	Pc	Pc	-	c	P	p		pc	c	p	p
**Chub mackerel (Cm)**	c	C	c	P	P	c	-				pc	c		
**Kingklip (Kk)**				c	c	c	c	-			c	c		
**Chokka (Ck)**	c			Pc	Pc				P		P	P	P	p
**Silver kob (Sk)**										-			c	c
**Snoek (Sn)**	c	C	c	c	c	c	c	c			-	pc	c	c
**Yellowfin tuna (Yf)**				c	c	c	c	c			c	-		
**Yellowtail (Yt)**										c	c		-	C
**Geelbek (Gb)**										c	c		C	-

## Results

The data included in analyses, after points outside of the known sampling area had been cleaned from the datasets, varied across the three time periods from several hundred survey intervals for pelagic surveys, to over three million hook sets for longline data (Table 3).

**Table 3:** Extent of the data included in analyses, averaged per year for Period 1 (1985–1991), Period 2 (1997–2000) and Period 3 (2003–2008). Units in which the effort data are originally recorded are shown.

Data source	Period 1	Period 2	Period 3	Units
**Pelagic survey**	549	669	909	Survey intervals
**Demersal survey**	8262	5564	8249	Trawl minutes
**Demersal commercial**	56107	43331	36912	Fishing hours
**Line fishery**	31809	29171	20326	Catch days
**Longline**		623780	3066716	Hooks set

**Fig 2:**
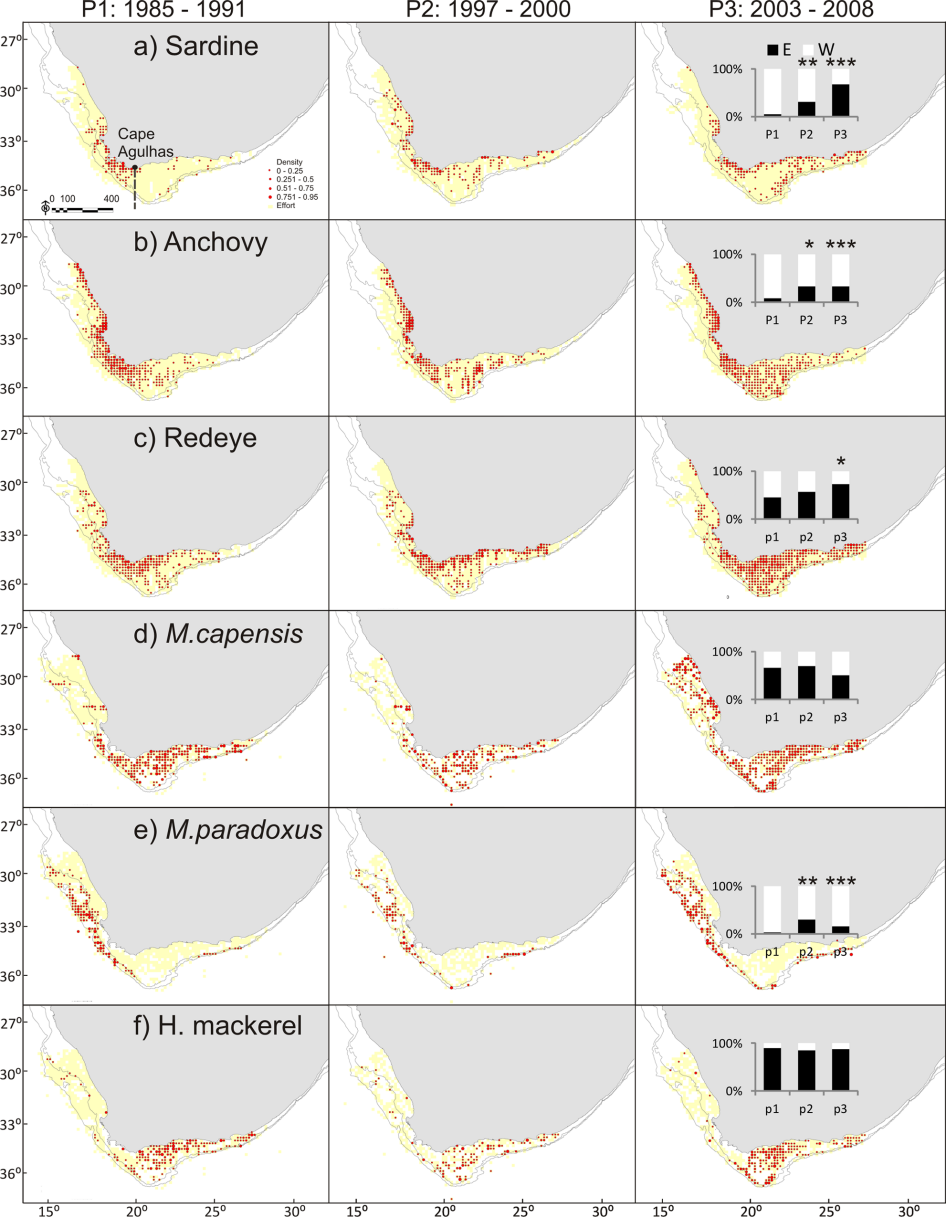
**a—f: Distribution maps for sardine, anchovy, redeye, *M*. *capensis*, *M*. *paradoxus* and horse mackerel during Periods 1–3.** The inset figure represents the proportion of biomass found east and west of Cape Agulhas in each period. Asterisks indicate where a significant change from P1 was detected on further statistical analysis, results below. * = p < 0.05; ** = p < 0.01; *** = p< 0.001.

**Fig 3:**
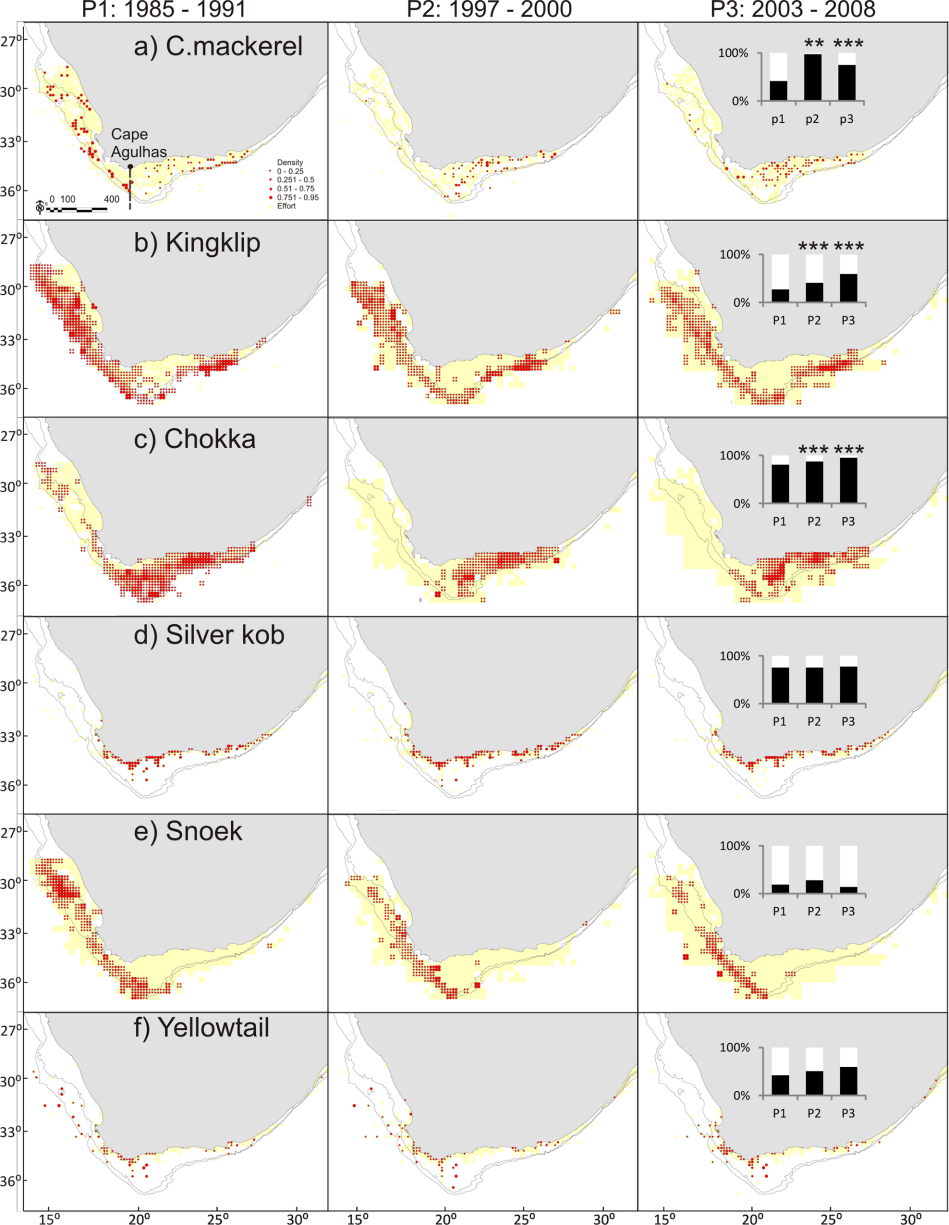
**a—f: Distribution maps for chub mackerel, kingklip, chokka squid, silver kob, snoek and yellowtail during Periods 1–3.** The inset figure represents the proportion of biomass found east and west of Cape Agulhas in each period. Asterisks indicate where a significant change from P1 was detected on further statistical analysis, results below. * = p < 0.05; ** = p < 0.01; *** = p< 0.001.

**Fig 4:**
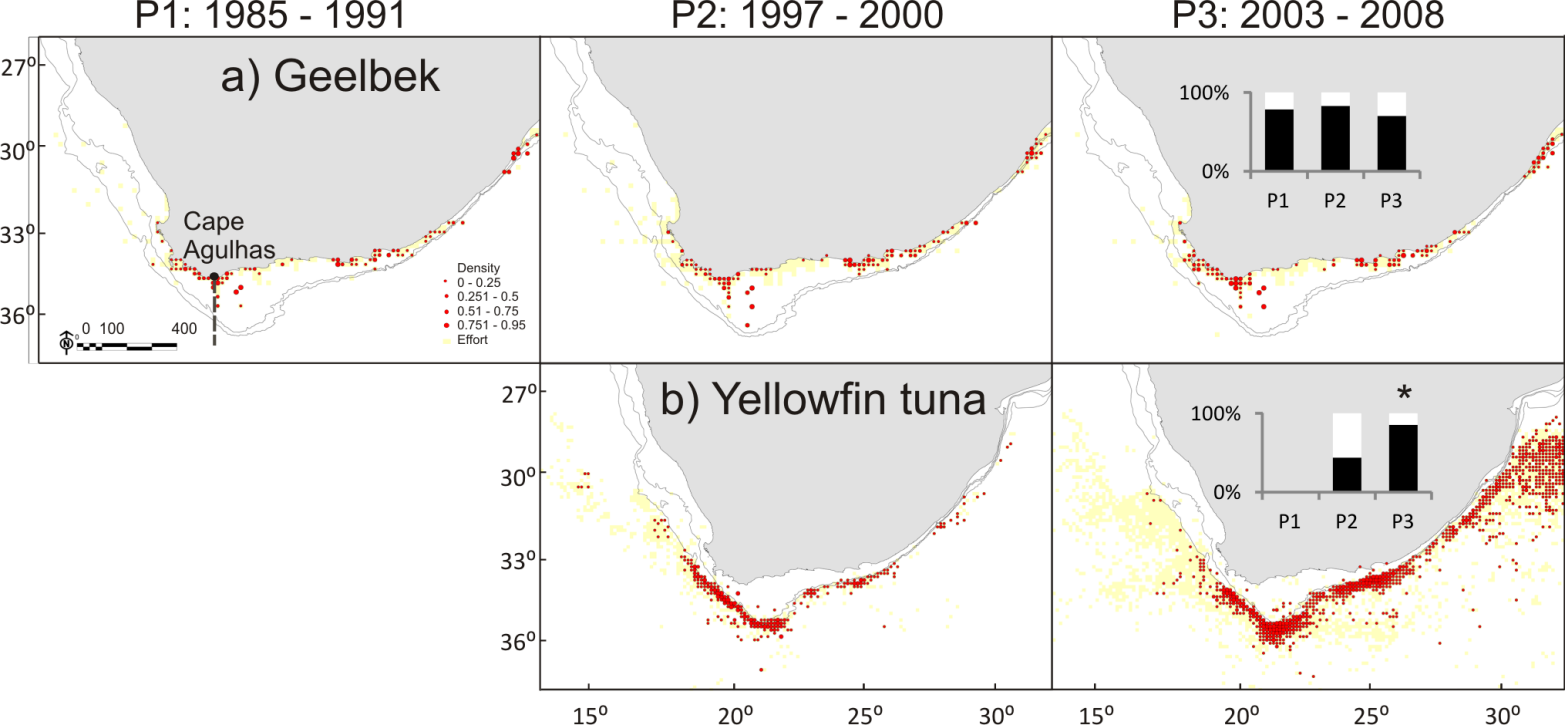
**a and b: Distribution maps for geelbek and yellowfin tuna during Periods 1–3.** The inset figure represents the proportion of biomass found east and west of Cape Agulhas in each period. Note there are no data for yellowfin tuna in Period 1 1985–1991. Asterisks indicate where a significant change from P1 was detected on further statistical analysis, results below. * = p < 0.05; ** = p < 0.01; *** = p< 0.001.

**Fig 5:**
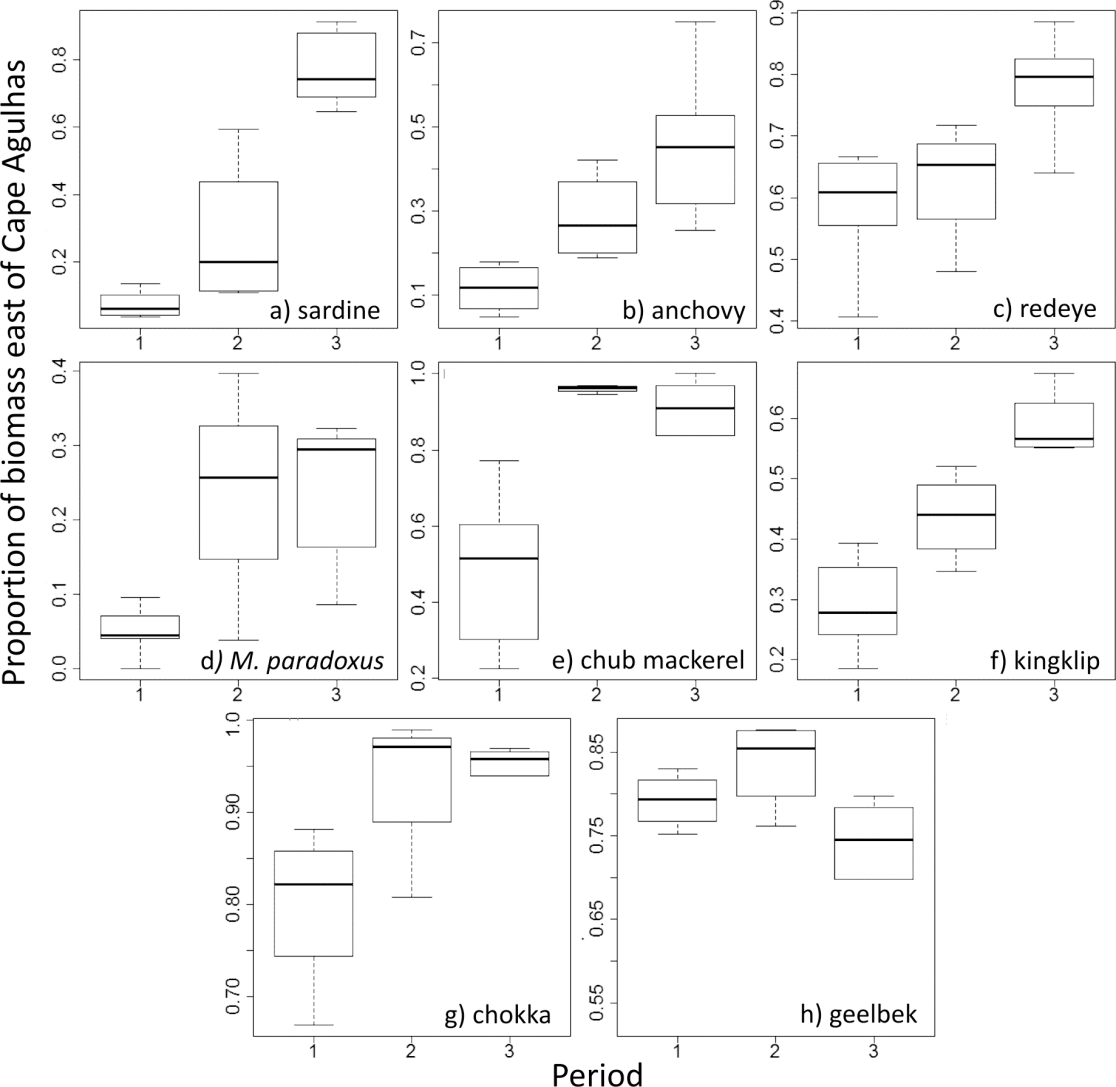
Estimated proportion of biomass east of Cape Agulhas in each period examined, for species where a significant change over time was found. Proportions were estimated using a beta regression model fitted to the observed proportions, with period as an explanatory variable. The median, upper quartile, lower quartile and interquartile range are shown.

Analyses, including results from beta regression GLMs, confirm the increase in proportion of sardine biomass east of Cape Agulhas when compared with Period 1 (P2: p < 0.01, P3: p < 0.001, pseudo R^2^ = 0.86) as previously illustrated by [50] and [51]. A similar but lesser pattern is also present in a number of other species, including kingklip (P2: p < 0.001, P3: p < 0.001, pseudo R^2^ = 0.79), anchovy (P2: p < 0.05, P3: p < 0.001, pseudo R^2^ = 0.6), chokka (P2: p < 0.001, P3: p < 0.001, pseudo R^2^ = 0. 57), *M*. *paradoxus* (P2: p < 0.01, P3: p < 0.001, pseudo R^2^ = 0.41), redeye (P3: p < 0.05, pseudo R^2^ = 0.32)) and chub mackerel (P2: p < 0.01, P3: p <0.001, pseudo R^2^ = 0.26); (Figs 2–5). Geelbek showed the opposite trend; the proportion found east of Cape Agulhas was significantly lower in Period 3 compared to Period 1 (p < 0.05. pseudo R^2^ = 0.42) (Figs 4 and 5). Not all changes over time were linear however. *M*. *paradoxus*, chub mackerel and snoek for example increased on the south coast from Period 1 to Period 2, and then declined again in Period 3, although *M*. *paradoxus* and chub mackerel both remained at significantly higher levels on the south coast in Period 3 than they had been during Period 1.

Horse mackerel, and the linefish kob, snoek and yellowtail all showed similar proportions on the west and south coasts over all periods examined. Although effort in the tuna longline fishery expanded between periods 2 and 3, only catches made in the commonly sampled area were considered, and a far higher proportion of biomass was on the south coast than the west (77%) in the later period.

**Fig 6:**
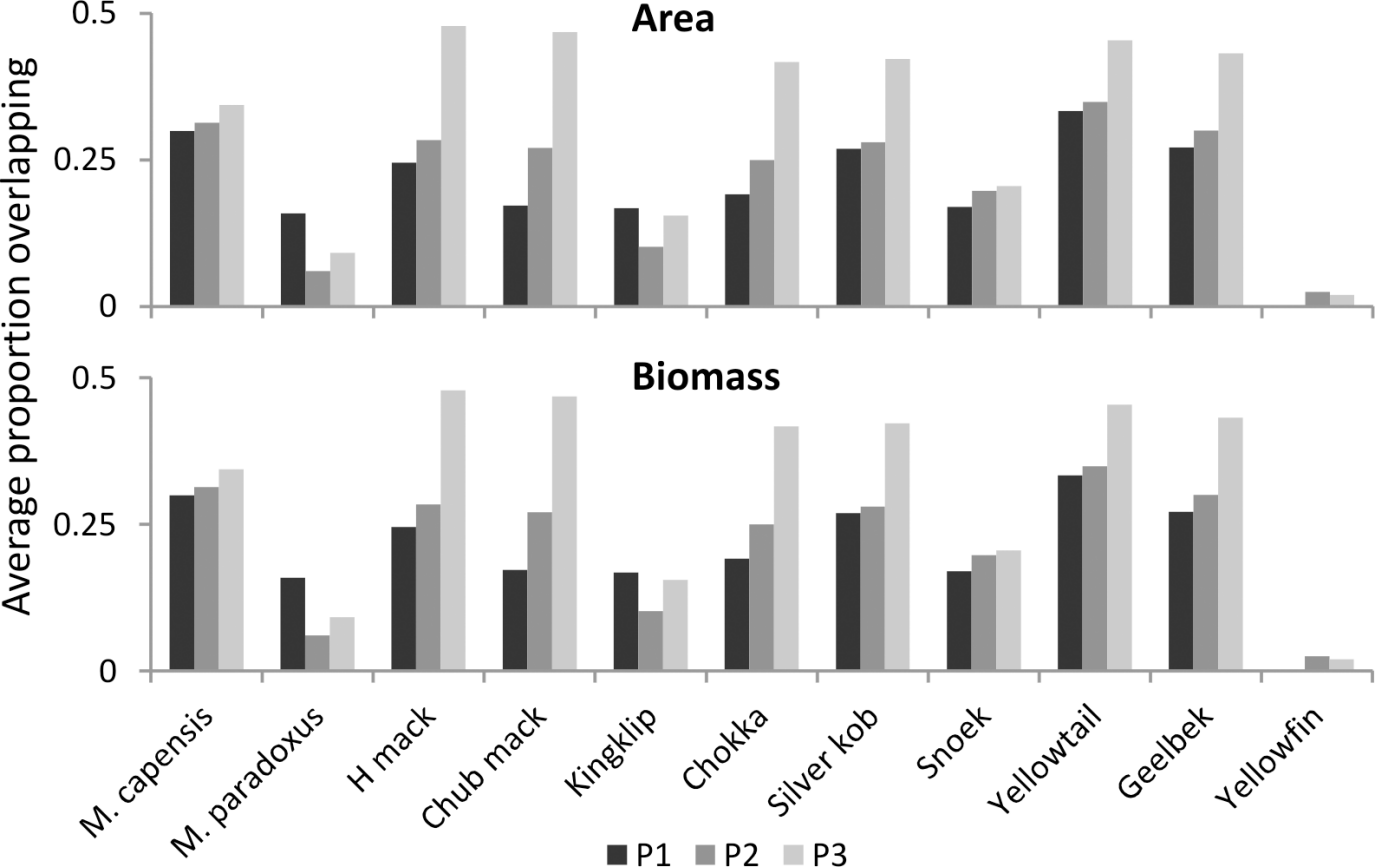
Average overlap in area and biomass of all other species with small pelagic fish species (sardine, anchovy and redeye) for the three periods examined.

**Fig 7:**
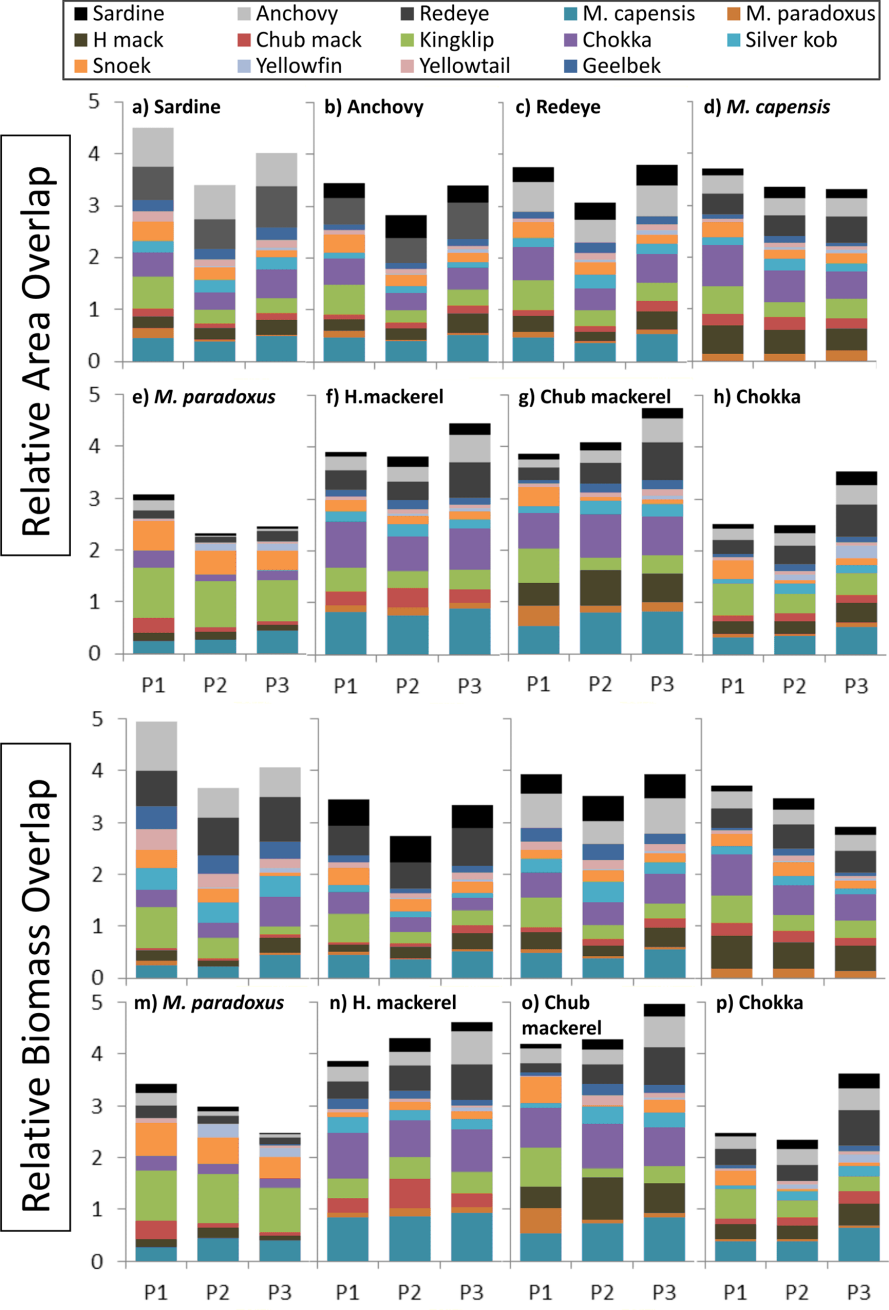
**a—p: Relative overlap in area and biomass between selected species and all other species mapped during each period.** 1 unit on the y-axis is equivalent to 100% overlap. For all overlaps by species and coast, see supporting information (S1–S5 Figs).

Similar patterns were illustrated by ROA and ROB (Figs 6 and 7) and there was increasing overlap between small pelagic fish as a group (including sardine, anchovy and redeye), and horse mackerel, chub mackerel, chokka, kob, yellowtail, geelbek, and to a lesser extent *M*. *capensis*, over all time periods (Fig 6). *M*. *paradoxus*, although displaying a slight increase in overlap with redeye, overlapped less with sardine and anchovy over time. On closer examination of results, this is a reflection of a decrease in overlap with small pelagic fish on the west coast (see the Appendix), where the majority of *M*. *paradoxus* biomass is found (Fig 2E). Overlap of both biomass and area with small pelagic fish increased over time on the south coast for *M*. *paradoxus* however, but this is masked by the west coast trend.

In general overlaps were lower during Period 2, increasing again in Period 3 (Fig 7), except in the case of the two hake species, which displayed similar levels in Periods 2 and 3 with regard to area overlap, while biomass overlaps declined over all three periods. All overlaps between each species, separated for south and west coasts, are illustrated in the Appendix, displayed between each species and east and west of Cape Agulhas.

**Fig 8:**
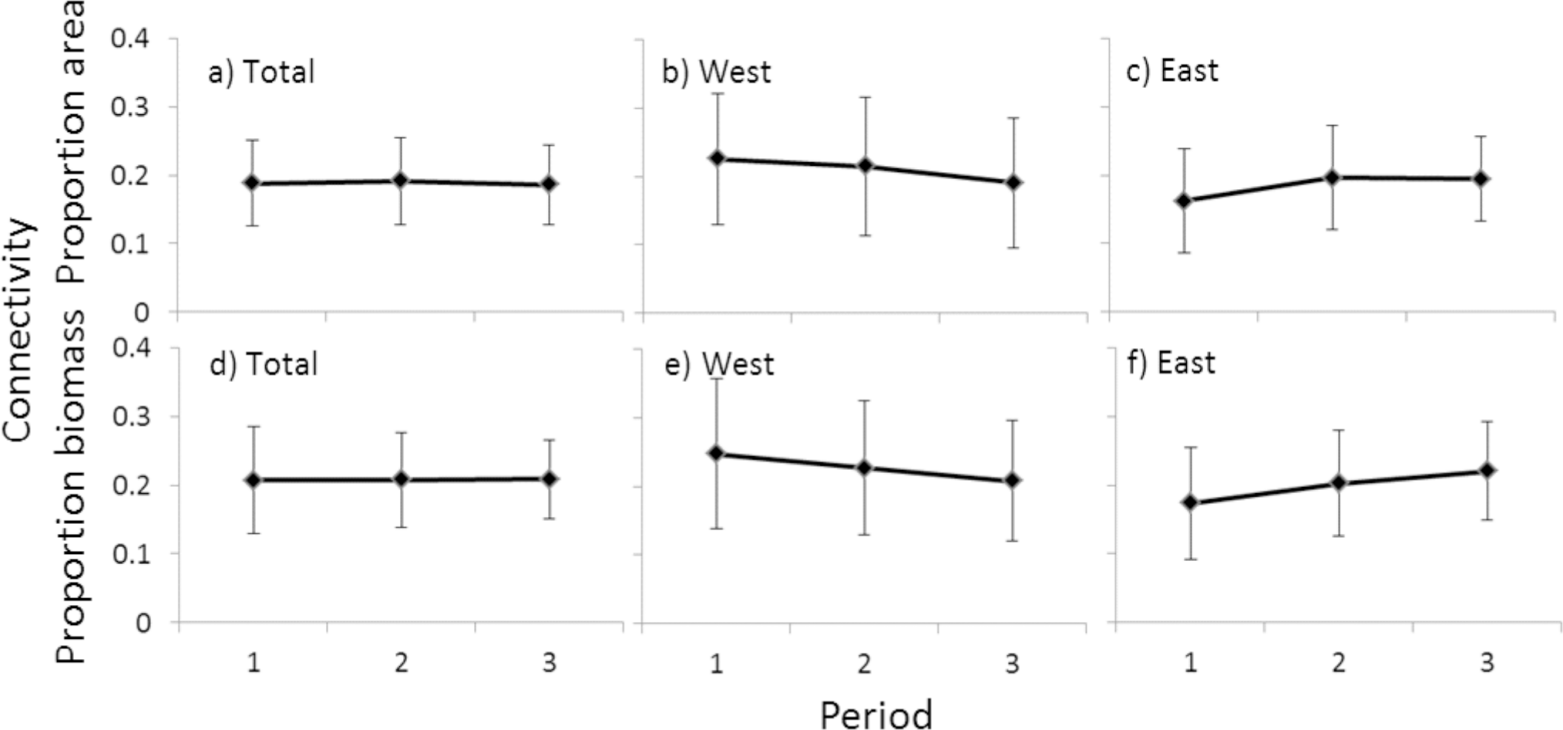
**Mean and standard deviation of system connectivity, or overlap in area (ROA) and biomass (ROB) between trophically related species** (see Table 2 for relationships), shown for the total system (a and d) and regions east (c and f) and west (b and e) of Cape Agulhas.

The pattern over time in system connectivity was similar based on area or biomass overlap (Fig 8), with a slight declining trend west of Cape Agulhas and increasing trend east of Cape Agulhas over time. When the total system is examined however no trend is detectable (Fig 8A and 8D), with modelled ROA and ROB also showing no significant difference from Period 1. Modelled predicted overlap in both area and biomass, east and west of Cape Agulhas was, however, significantly different in Periods 2 and 3 when compared with Period 1 (p < 0.0001), but in all cases models had low explanatory power (pseudo R^2^ = 0.023–0.029).

**Fig 9:**
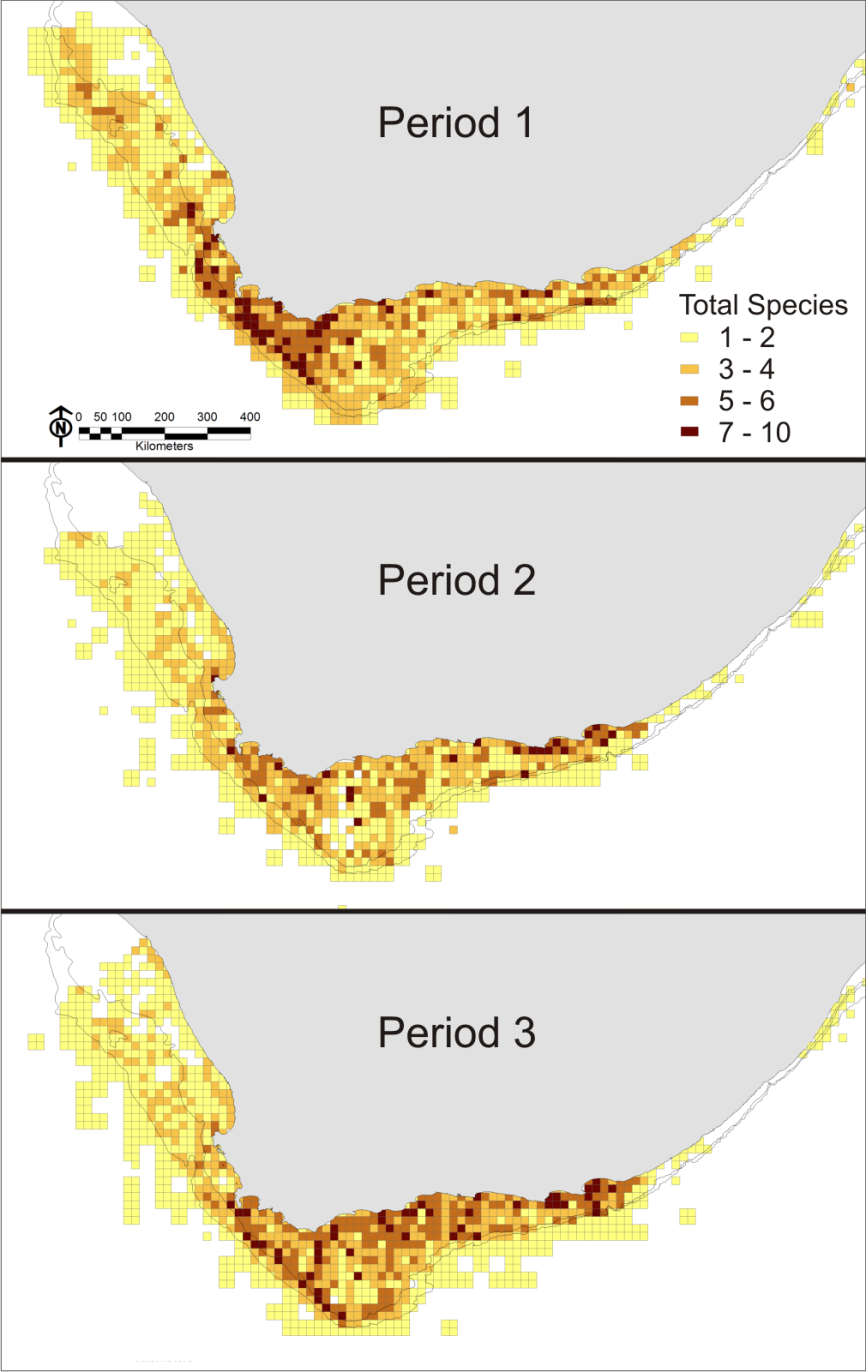
The number of the 13 mapped species found per grid cell during each period. Yellowfin tuna were excluded from this analysis due to a lack of data in period 1.

**Fig 10:**
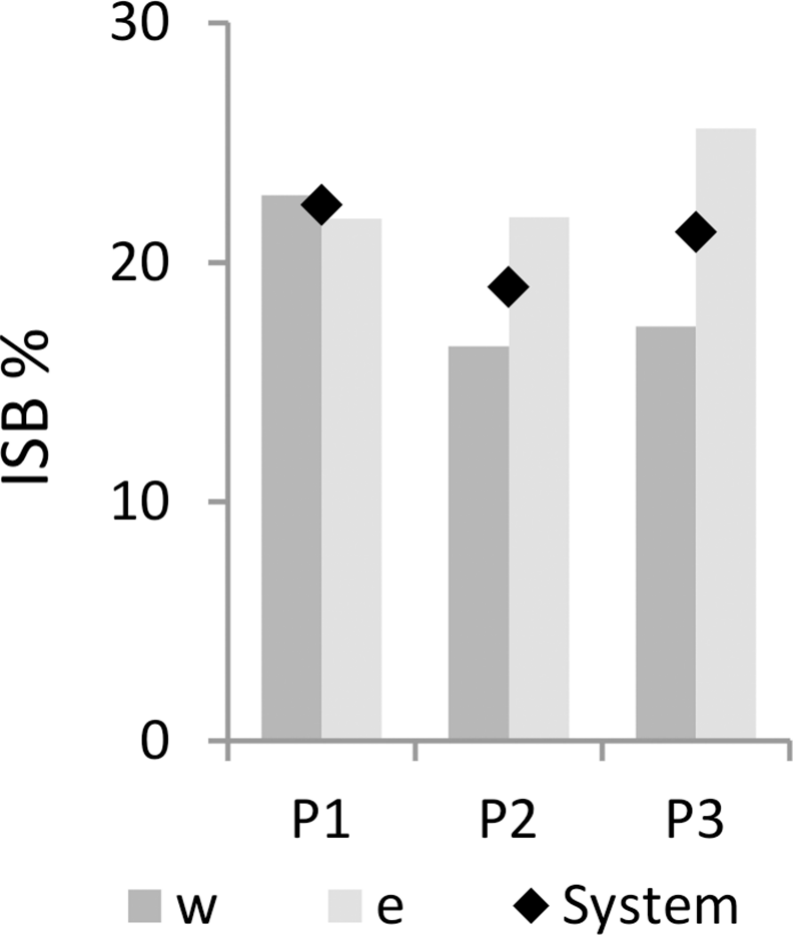
Index of spatial biodiversity (ISB) east and west of Cape Agulhas, and for the system as a whole, during periods 1–3.

Increasing diversity on to the east when compared with the west coast is illustrated both by the maps of the combined species distributions (excluding yellowfin tuna due to lack of data for Period 1) (Fig 9), and by the Index of Spatial Biodiversity, ISB. The index was similar around the coast during Period 1, but over time decreased in the west and increased in the east (Fig 10). When data were used to fit beta regression GLMs, overall ISB (ISB_system_) was significantly lower for both Period 2 (p < 0.01) and 3 (p < 0.001) when compared with Period 1, although the explanatory power of the model was low (pseudo R^2^ = 0.01). The same results were true for the west coast as well (both p < 0.001, pseudo R^2^ = 0.04). ISB east of Cape Agulhas was not significantly different in Periods 2 and 3, but the model had an even lower explanatory power (pseudo R^2^ = 0.007).

As in [30], ISB (ISB_all_) was also calculated based on all common species identified by demersal surveys during each period (161 spp.) and compared to the above findings based on 13 species. As expected due to the large number of species included, ISB_all_ was much lower than when calculated based on the 13 mapped species included, being lowest in Period 1 (8.93%), increasing to approximately 11% in Periods 2 and 3. In all periods ISB_all_ was approximately 20% higher in the area east of Cape Agulhas than on the west coast.

**Fig 11:**
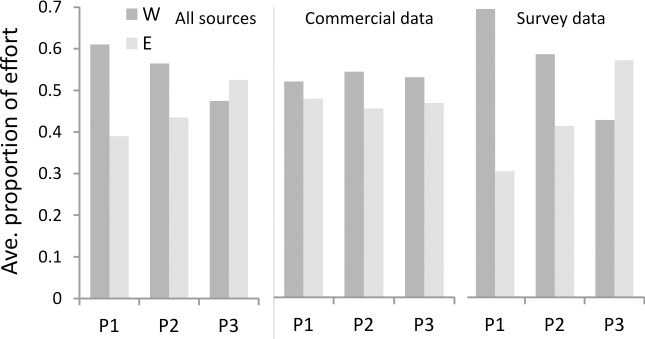
Proportion of effort east and west of Cape Agulhas averaged for all data sources used to construct distributions. Effort is shown both combined from all sources, and for commercial and survey data separately, during periods 1–3 (P1 –P3).

Effort from all data sources increased on the east coast over time and decreased on the west (Fig 11). Due to the initial survey-driven bias towards the west coast, this has meant effort in the most recent period (3) is the most evenly distributed by coast of the periods examined. This difference is driven by increased survey effort rather than increasing commercial effort on the east coast (for example proportion of demersal commercial effort on the east coast in Period 1: Period 3 is 56:59%, while that for demersal survey is 33:62%).

## Discussion

The pattern of eastward movement highlighted in this study reflects what has already been illustrated for both sardine [50,51] and anchovy [15,31], but is not consistently present in all higher trophic level species as one might expect. Although both hake species are strongly trophically linked to various other species that display increased abundance on the south coast over time (Table 2), e.g. small pelagic fish, horse mackerel and chokka, neither has followed the same pattern. The proportion of *M*. *capensis* on the south coast did not increase significantly in Period 2, and proceeded to decline in Period 3 while the proportion of *M*. *paradoxus* on the other hand was significantly higher on the south coast during Periods 2 and 3. The relationship to changes in sardine was not linear however, with a decline in prevalence on the south coast between Periods 2 and 3. If dietary prevalence of small pelagics or chokka is taken into account, kob, geelbek and yellowtail would also have been expected to exhibit a parallel increase on the south coast. On the contrary, geelbek decreased in abundance in the east during Period 3. Yellowtail did display an increasing but non-significant trend. While kingklip did increase proportionally east of Cape Agulhas over time, it is possible that this is related to successful implementation of management actions in the region, such as the ‘kingklip box’ seasonal closure near Port Elizabeth [52], rather than a shift in distribution.

Although various non-climate factors, such as the recovery of previously exploited stocks in the east, should be considered as possible explanations of the increase in abundance in this area, in this case they do not provide the most likely cause. Overall, fishing effort on the south coast has not declined over the periods examined (Fig 11). The main species group displaying this increase in easterly abundance–small pelagic fish–has been both relatively extensively fished and well monitored by biannual surveys, as described in the methods section. Neither survey data (used to construct these distributions) nor catch data for those species reflect any heavy exploitation and subsequent recovery in the east. They do however support the hypothesis that sustained effort on the west coast concurrent with declines in biomass in that region may have exacerbated the pattern [50]. In the line fishery, heavy depletion of silver kob and geelbek stocks along with other targeted species by the late 1990s [40] did lead to a declaration of a state of emergency in the fishery, and the development of the linefish management protocol [53] (an approach using biological reference levels for management planning). Although the resultant reduction in effort by approximately 70% in 2000 was substantial, and there is an indication that silver kob catch per unit effort may be increasing [54], this is not reflected in results presented here and the status of most line fish species remain collapsed or unknown [9]. Pressure from the line and inshore trawl fisheries remains relatively high given the low abundance of these species [41,55], and is still likely to effect the ability of line fish species to respond to increases in prey abundance. Changes in demersal assemblages over the period examined are also not thought to be linked only to changes in fishing effort [32]. As a result, and given the widespread nature of the shifts observed here as well as described elsewhere [9,14], relaxation of effort leading to a regional recovery is not a probable explanation and system-level climate forcing seems likely to have played a major role.

Top predators were not included in this study, however, as previously discussed, effects of this change in small pelagic fish distribution on seabirds have been well documented [19,21,56]. Although the prevalence of sardine in the diet of Cape fur seals, opportunistic top predators, has been shown to reflect local availability, no concurrent change in seal populations with changes in small pelagic fish abundance or distribution has been recorded in South Africa [57]. This stable distribution is largely attributed to the lack of additional suitable breeding habitat limiting any potential expansion of the population [58, 59]. The decline and subsequent expansion of populations at the northern extent of their range in Namibia and Angola during the 1990s and early 2000s have been linked to changes in abundance and distribution of prey species and supports this hypothesis, illustrating a direct effect on seals due to changes in prey availability if space limitation is not a factor [58]. An additional factor dampening possible seal distributional response is the opportunistic nature and omnivory of seals [57,59].

A number of species show peaks in abundance east of Cape Agulhas during Period 2, along with the documented shift in anchovy spawner biomass and eggs east of Cape Agulhas [15]. This abrupt change in anchovy distribution has been linked to environmental changes, possibly induced by increased coastal upwelling east of Cape Agulhas and the resultant improvement of feeding conditions for anchovy spawners relative to those on the west coast [31]. This may illustrate an environmentally-mediated, bottom-up mechanism of trophic control whereby changes in primary production affect higher trophic level abundance and distribution [60]. The same mechanism may have influenced the distribution of other species shown here to have increased on the south coast during Period 2, (e.g. chub mackerel). Because the upwelling in question is restricted to coastal regions, however, it is unlikely to have directly effected the Period 2 increase in snoek east of Cape Agulhas, where records are largely further offshore.

Although plotting only 95% of the distribution has been suggested as too low a threshold [24,29], in this context where a map based on a single data source was used to explore change over time, it was deemed a reasonable if conservative measure, allowing for meaningful interpretation of overlaps. As expected, where both species in the pair being examined for overlap exhibited an increase in proportion on the south coast, degree of overlap increased. In many cases, the trophic functioning of the system is likely to have been affected by changes in overlap since trophically related species (see Table 2) are not sharing the same proportions of their distributions as they were previously. Chokka squid, for example, is more prevalent on the south coast. The degree of overlap with a species increasingly found on the south coast, e.g. sardine, will thus have increased. As both predation and competition relationships exist between the two species (Table 2), trophic interactions within the region must be altered. However, that species pairs exhibiting increased overlap are not always coupled predator and prey species seems to imply an outside driving force, or drivers, other than trophic interactions considered here, such as environmentally favourable conditions to the east as discussed by [31].

System connectivity (Fig 8) can give an indication of the ecosystems resilience and ability to withstand change. While the connectivity of the system as a whole does not appear to have changed (Fig 8A and 8D), the decline in connectivity in the west (Fig 8B and 8E) and the increase in connectivity in the east (Fig 8C and 8F) may depict the rise in trophic importance of the south coast since Period 1 as more potential trophic interactions are located there than on the west coast. Connectivity in this study has been based on the interaction among only the 14 species examined and known to be trophically linked (Table 2) and is thus not truly a reflection of the whole ecosystem. It does however, include the most prevalent, commercially and trophically important species, allowing for comparison between time periods.

Despite its drawbacks, the ISBj indicator is an appropriate measure to have used in this analysis where the overarching, within-system change is of interest. ISBj does seem to reflect the increasing food web complexity on the south coast that has been suggested by increasing connectivity on that coast. The inclusion of a large number of less prevalent species in this calculation of ISB_all_ (not pictured in Fig 10) gives an overview of system state from a different perspective, but also explains the lack of agreement between the outcomes of the two ISB calculations (a dip in ISB_j_ in Period 2, and an increase in ISB_all_ from Period 1 to Periods 2 and 3).

The different data sources used have their own advantages and disadvantages. Survey data are by definition more suited to produce an accurate index of biomass, although it should be kept in mind that demersal surveys have been designed around hake and the pelagic surveys around anchovy and sardine, thus are not as representative for other species. For example, both redeye and chub mackerel are likely to have ranges extending further offshore than is captured by surveys., and horse mackerel have been recorded by acoustic survey over the shelf-break area when demersal survey methods failed to detect them there [61]. Commercial data on the other hand, while providing far greater sampling effort than surveys can, are unable to provide unbiased data as the effort is not random. Commercially viable concentrations of target species are actively sought out, for example data for the pelagic fishery will reflect only those fish that are accessible in terms of port and processing facilities. Commercial data can potentially provide more accurate information and better coverage for species that are not the object of survey data collection, but the limitations must be kept in mind when assessing results. Unfortunately, any single data source is very unlikely to represent the full extent of a species distribution. The method can, however, still provide a useful means of comparing change over time, as has been done here, rather than reporting absolute distributions.

Results could be improved if the disparate distributions and trophic roles of juveniles and adults of trophically important species could be taken into account. These data are only available at an ecologically satisfactory level of detail for sardine and anchovy (although data for redeye are also available, they do not cover the entire distribution). The decision to combine the May and November recruit and spawner biomass survey data for the small pelagic fish species was based on the goal of investigating ecosystem structure and functioning, and as such the need to understand the availability and distribution of small pelagic as potential food represented by all life-stages of a predatory species within the ecosystem. However, the differences between the recruit and spawner distributions identified by each survey should be kept in mind. For example, the eastward distributional shift in anchovy [15] is evident only in the spawner biomass, while recruits remain almost entirely on the traditional west coast nursery grounds (see supporting information S6 Fig), with greater implications for the seasonality of prey availability to predators. On the other hand, sardine and redeye display similar trends in both recruit and spawner biomass distributions. Both increasingly are found east of Cape Agulhas over time, with redeye recruits actually showing a more pronounced distributional trend towards the east than displayed by the redeye spawner biomass data (37% vs 18% increase in proportion found east of Cape Agulhas from Period 1 to Period 3). Thus, while sardine and redeye seem to have undergone a distributional shift, anchovy have experienced a shift in spawning area, and this must be taken into consideration when interpreting results.

In many cases distributions and patterns of interaction have changed over time. These changes and changes reported in top predator species such as seabirds support the idea that an understanding of trophic relationships is important if potential system-wide changes are to be understood or anticipated. Although previous studies based on ecosystem models have described changes to overall system functioning [62,63], patterns of change identified were not as strong when considering the average state of the ecosystem as a whole. Regional differences between the areas east and west of Cape Agulhas were evident, however, with notable shifts in species distributions and the potential interactions between species (predators, prey and competition for common prey). During Period 1, a number of species displayed a far greater prevalence on the west coast compared to their Period 3 distributions. Connectivity was highest on the west coast during Period 1, and to the east in Period 3, with an overall dip during the intermediate Period 2, a pattern largely echoed by the index of spatial biodiversity. Although unfortunately beyond the scope of this study, future expansion of this work to investigate links with physical variables would be an important step in understanding system function. Spatio-temporal variations in biomass and structural complexity clearly affect the structure and functioning of the system, and further understanding of the implications of changes such as those described here is important when attempting to appreciate the possible implications of current and future system-level change for the ecosystem and management of the fisheries it supports.

## Supporting Information

S1 Fig Overlap in area (ROA) and biomass (ROB) between sardine, anchovy and redeye and all other species, east and west of Cape Agulhas. (TIF)

S2 Fig Overlap in area (ROA) and biomass (ROB) between *M*. *capensis*, *M*. *paradoxus* and horse mackerel, and all other species, east and west of Cape Agulhas. (TIF)

S3 Fig Overlap in area (ROA) and biomass (ROB) between chub mackerel, kingklip and chokka squid, and all other species, east and west of Cape Agulhas. (TIF)

S4 Fig Overlap in area (ROA) and biomass (ROB) between Silver kob, snoek and yellowfin tuna, and all other species, east and west of Cape Agulhas. (TIF)

S5 Fig Overlap in area (ROA) and biomass (ROB) between yellowtail and geelbek, and all other species, east and west of Cape Agulhas. (TIF)

S6 Fig Average distribution of sardine, anchovy and redeye recruits (May) and spawner biomass (Nov) during each period, based on pelagic survey data. (TIF)
