# Supplementary figures and images for: Patterns of Distribution and Spatial Indicators of Ecosystem Change Based on Key Species in the Southern Benguela
Source: PLoS One. 2016 Jul 21;11(7):e0158734. doi: 10.1371/journal.pone.0158734 (PMC4956041; doi:10.1371/journal.pone.0158734)

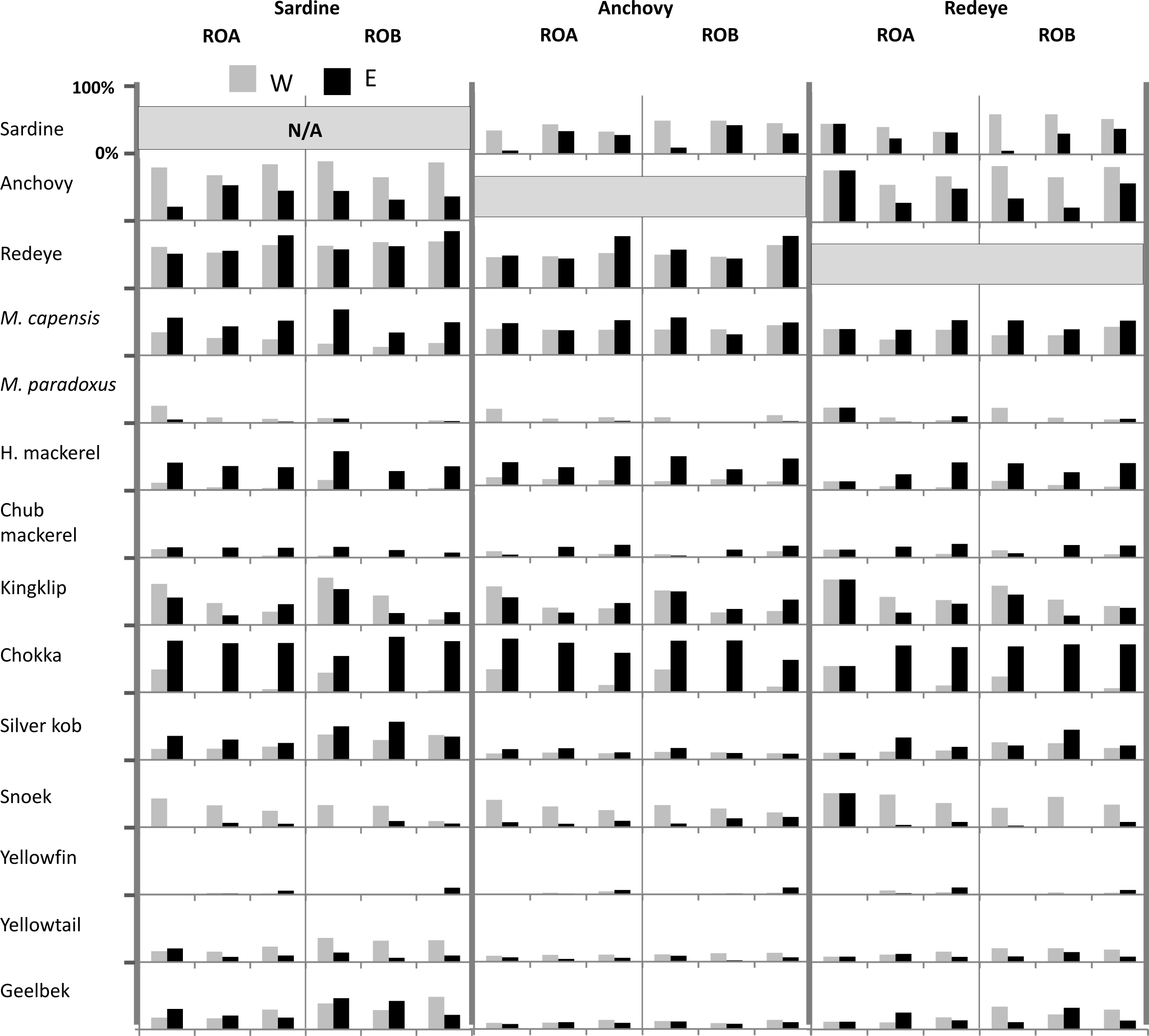

Supplement: S1 Fig — (TIF) [file pone.0158734.s001.tif]

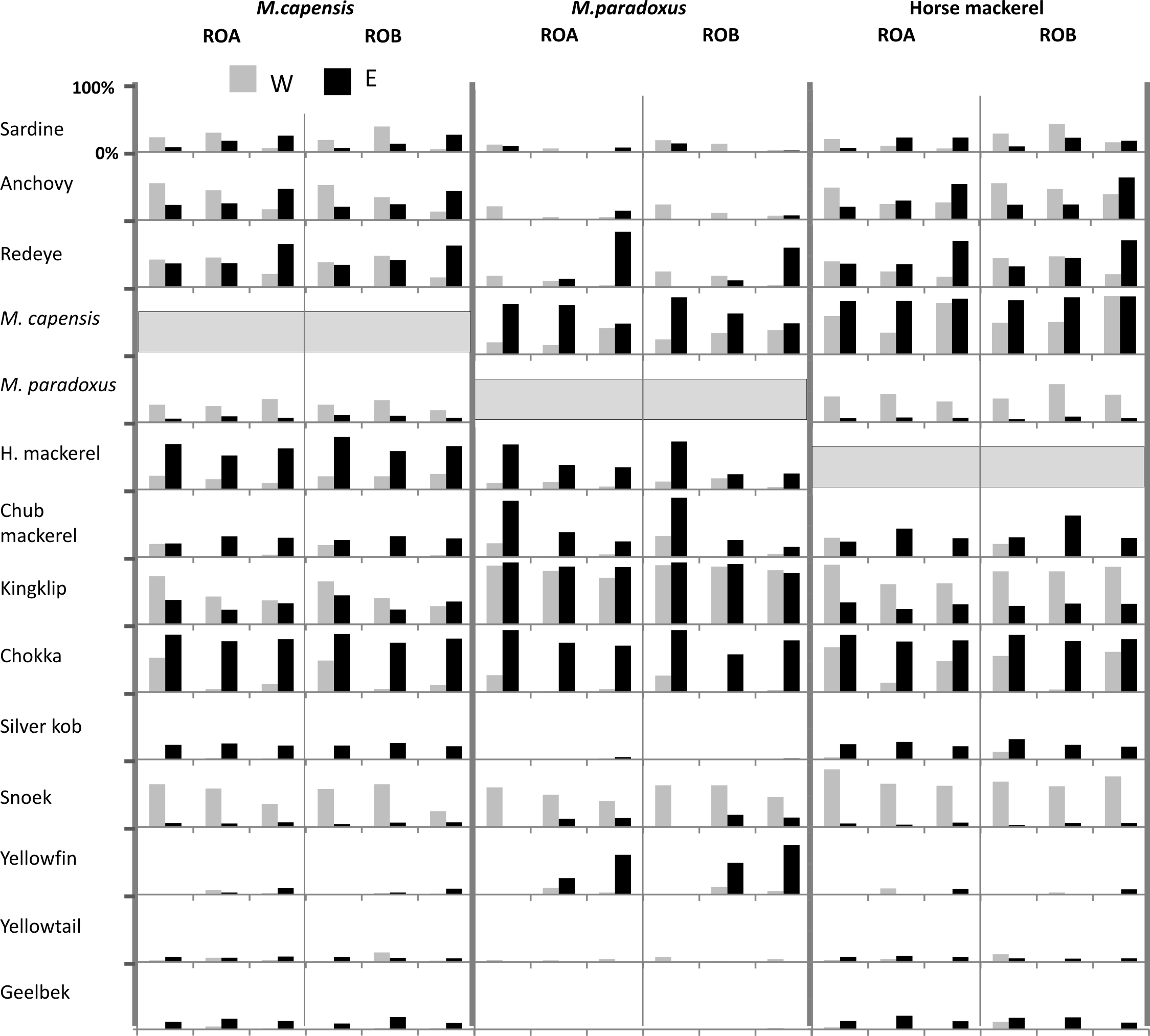

Supplement: S2 Fig — (TIF) [file pone.0158734.s002.tif]

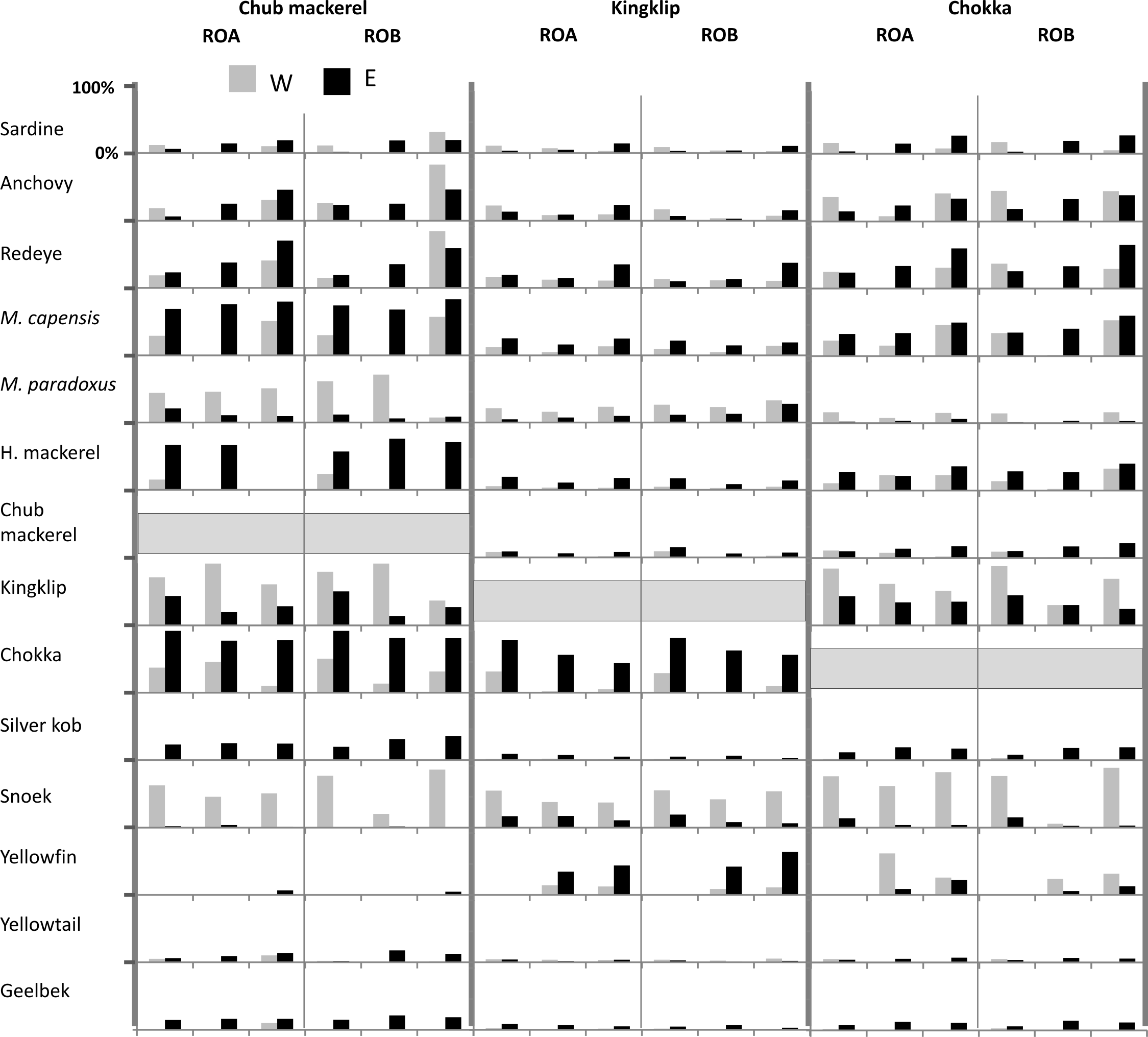

Supplement: S3 Fig — (TIF) [file pone.0158734.s003.tif]

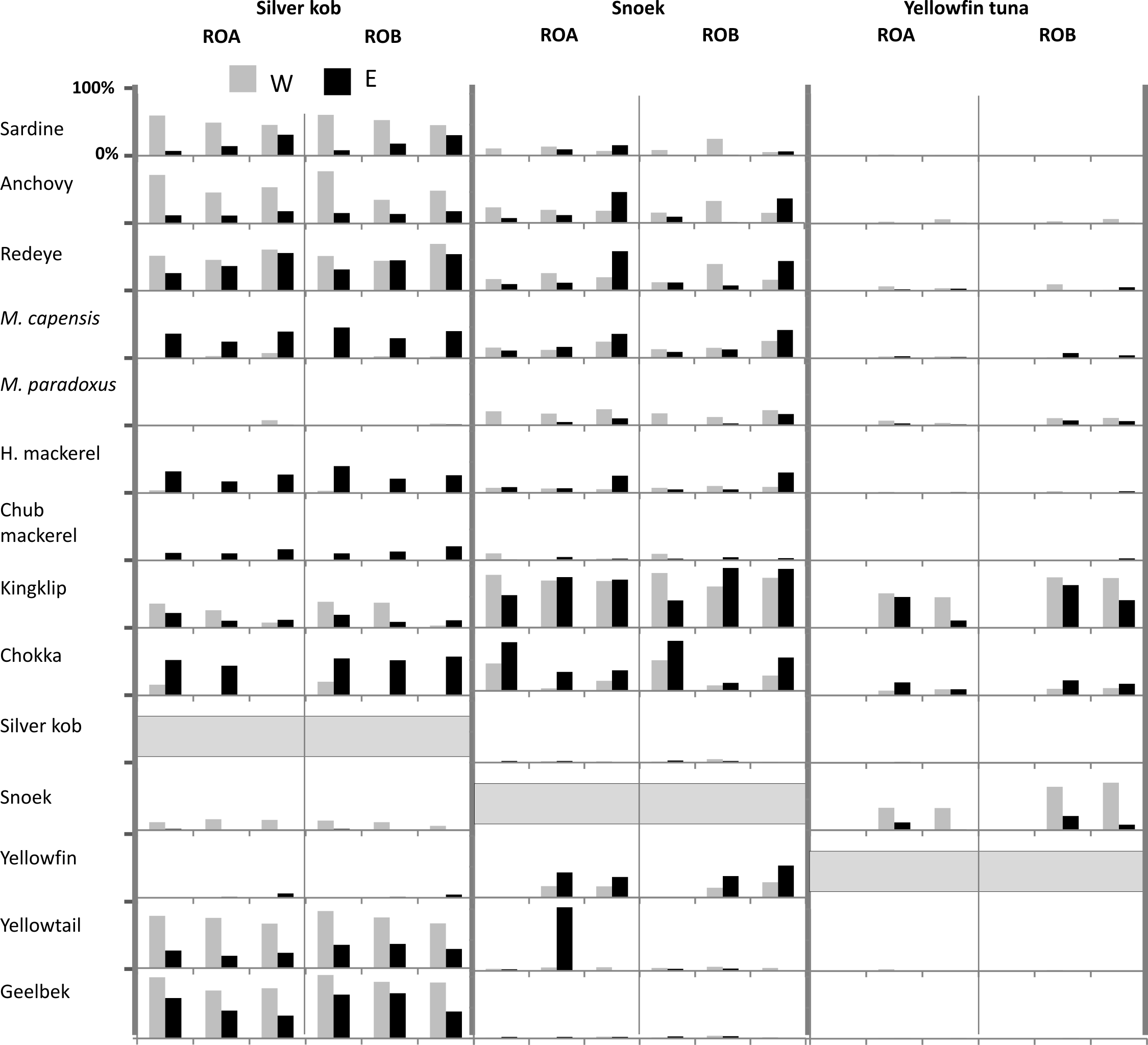

Supplement: S4 Fig — (TIF) [file pone.0158734.s004.tif]

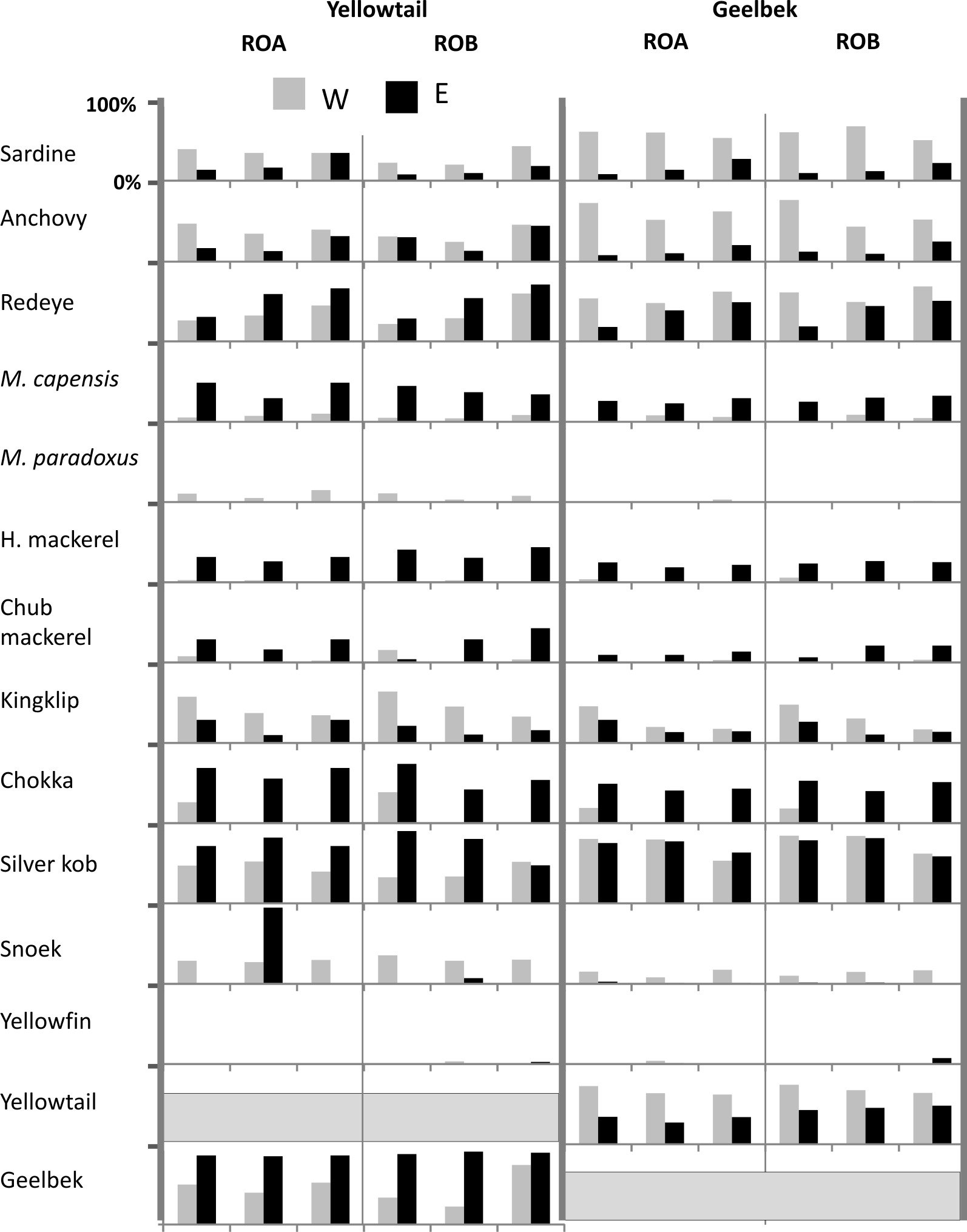

Supplement: S5 Fig — (TIF) [file pone.0158734.s005.tif]

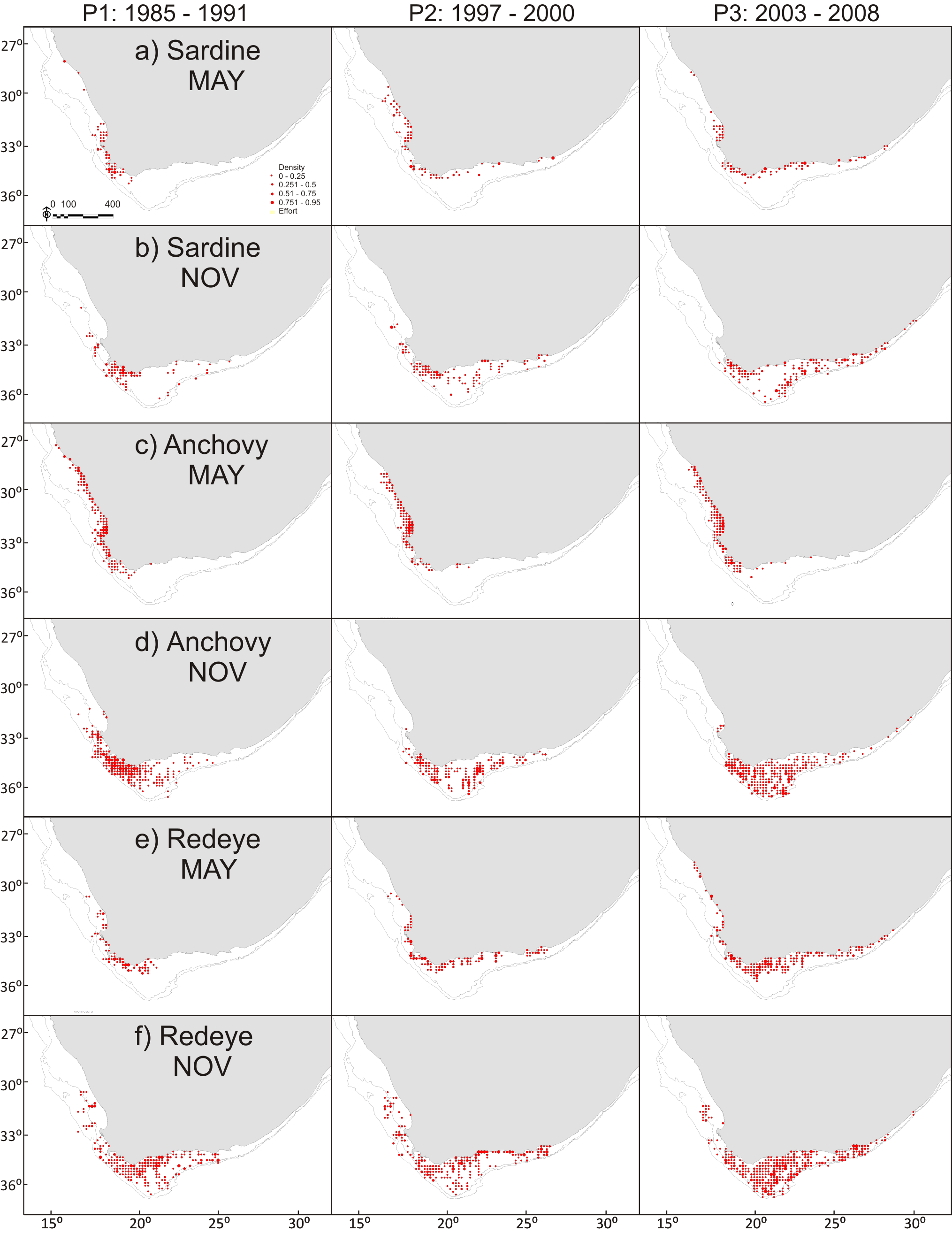

Supplement: S6 Fig — (TIF) [file pone.0158734.s006.tif]
